# Depression in military medicine cadets: a cross-sectional study

**DOI:** 10.1186/s40779-015-0058-x

**Published:** 2015-11-10

**Authors:** Dimitrios Nasioudis, Leonidas Palaiodimos, Matthaios Dagiasis, Angeliki Katsarou, Evangelos Ntouros

**Affiliations:** Department of Gynaecology, 401 Army General Hospital, Athens, Greece; Department of Medicine, Jacobi Medical Center/Albert Einstein College of Medicine, New York, USA; Naval Hospital of Athens, Athens, Greece; 251 Hellenic Air Force Hospital, Athens, Greece; Hellenic Military School of Combat Support Officers (SSAS), Thessaloniki, Greece

**Keywords:** Depression, Military medicine, Medical students, Medical education, Military training

## Abstract

**Background:**

Military medicine cadets undergo strenuous military training alongside demanding medical studies. This stressful and complex educational environment can lead to the emergence of depressive symptoms. We investigated the prevalence of depressive symptoms in a cohort of military medicine cadets.

**Methods:**

We conducted a descriptive questionnaire-based cross-sectional study among Greek military medicine cadets in the undergraduate program of the Hellenic Military School of Combat Support Officers. The Greek translation of the Zung self-rating depression scale questionnaire was used to screen for the presence of depressive symptoms. In addition, demographic, academic and dietary information was collected. The Shapiro-Wilk test of normality, Pearson correlation test, Chi-square test, *t*-test and Mann Whitney *U* test were employed for statistical analysis.

**Results:**

We enrolled 55 female and 91 male military medicine cadets with a mean age of 19.84 years (SD = 0.99). The mean Zung crude score was 43.32 (SD = 4.55): 42.8 (SD = 4.43) for female cadets and 43.64 (SD = 4.6) for male cadets. Cadets were further subdivided into low and high risk groups for the presence of depressive symptoms. We identified 57 (39 %) cadets with a total Zung crude score of 45 or above: 21 females and 36 males. Statistical analysis did not reveal any significant differences between the two groups based on gender, year of training, academic performance, alcohol consumption, smoking status, vitamin supplementation, dietary habits or BMI.

**Conclusions:**

We report a high prevalence of depressive symptoms in a cohort of military medicine cadets that underscores the need for effective screening and appropriate and timely interventions. We did not identify any related risk factors. Military medicine cadets are exposed to a challenging military and medical training environment, and thus represent a group at risk for development of depression.

## Background

Undoubtedly, medical training is a stressful process which can lead to the emergence of depressive symptoms [1]. The military environment, especially during strenuous military training, may also be characterized by a high prevalence of depressive symptoms [2]. Psychiatric conditions remain underdiagnosed, both in medical professionals, including medical students, and in military personnel; members of these groups are often reluctant to seek professional help [3, 4]. Multiple cross-sectional studies examining the point prevalence of depressive symptoms among medical students during their medical education have reported rates that range from 2.9 % to 50 % [3, 5–7]. In these studies, academic performance, year of training and gender have been associated with the presence of depression [3, 5–7]. A personal history of formally diagnosed depressive episodes or a history of self-defined depressive episodes is strongly associated with higher point prevalence of depressive symptoms [3, 6].

However, there is a paucity of evidence regarding the prevalence of depressive symptoms in military undergraduates [8]. Depression may have a major negative impact on the military performance and personal life of cadets, leading to alcoholism, substance abuse, attrition and suicide [9–13]. Consequently, it is essential to ensure early diagnosis, adequate access to psychiatric care and prompt treatment. In conducting this cross-sectional study, we aimed to investigate the prevalence of depressive symptomatology among military medicine cadets in our academy, evaluate differences in their lifestyle characteristics and examine possible risk factors.

## Methods

This cross-sectional study was conducted at the campus of the Hellenic Military School of Combat Support Officers (Greek Spelling: Stratiotiki Scholi Axiomatikon Somaton, SSAS) in Thessaloniki, Greece, in March 2013. Briefly, military medicine cadets complete a 6-year long medical curriculum provided by the Aristotle University of Thessaloniki Faculty of Medicine. In addition, they reside within the campus of SSAS, where they receive intense military training in the form of basic combat training, compulsory military courses and an introduction to military life and discipline. All candidates applying to the Hellenic military academies are pre-screened before admission for major psychiatric conditions. The psychiatric assessment of each candidate includes the administration of psychometric instruments such as the Symptom Checklist- 90- R and the Minnesota Multiphasic Personality Inventory (MMPI) followed by an interview conducted by a psychiatrist and a psychologist; candidates with an active Axis I (DSM-IV) pathology, including current depression or a personality disorder (Axis II), are excluded from service. History of depressive episodes when disclosed by the candidate is also a criterion for exclusion.

The current study was based on data provided voluntarily by 146 military medicine cadets in the primary 3 years of the undergraduate program; 3 cadets chose not to participate. After a detailed presentation of the project, verbal informed consent was obtained and a paper-based questionnaire was distributed to all participants; each participant was asked to provide basic demographic data including gender, age, year of training, height, and weight. Cadets were also asked to provide information concerning their academic performance, smoking status, and dietary habits (daily fruit/vegetable/coffee consumption, fish servings consumed weekly, use of multivitamin supplements, and regular consumption of energy drinks). Reported height and weight were subsequently used to calculate BMI values. All data in our study were collected via self-report and were not verified against more objective data sources. The study protocol and administered questionnaires were reviewed and approved by the appropriate committee of SSAS; our study complied with the ethical principles for medical research involving human subjects, as outlined in the Declaration of Helsinki.

Regarding key demographic characteristics of the study population, we enrolled 55 (37.7 %) female and 91 (62.3 %) male cadets, with a mean age of 19.84 years (SD = 0.99). Thirty-six (24.7 %) cadets were in their first year of training; 50 (34.2) and 60 (41.1 %) were on their second and third year of training, respectively.

Simultaneously, the Greek translation of the Zung self-rating depression scale questionnaire was administered. The Zung self-rating depression scale is a 20-item scale that covers affective, psychological, and somatic features of depression. It has already been translated and validated in the Greek general population, displaying good reliability and validity (both sensitivity and specificity exceeded 90.00 at a cut-off of 44/45, with Chronbach’s alpha and the Pearson coefficient equal to 0.09 and 0.92, respectively) [14]. All elements of the survey were completed anonymously in an attempt to ensure honesty in the cadets’ responses. We also protected anonymity by not collecting any identifying information, such as name, address, email address or date of birth. Statistical analysis was performed using the SPSS® (v17.0) statistical package, and alpha value of statistical significance was set at 0.05.

## Results

Following the collection of data captured through the aforementioned questionnaires, cadets were subdivided into three groups based on their academic performance compared to that of their peers, after taking into consideration their grade point average (GPA) and number of failed courses. In our academy, a GPA that is one standard deviation above or below the mean of the class is considered as high or low academic performance, respectively. As such, the average academic performance group comprised 68 % of the total population (*n* = 91), whereas high and low academic performance groups each were 16 % of the cadet population (*n* = 27 and *n* = 28, respectively). Two groups were formed on the basis of alcohol consumption. The first group included cadets who never drink or have one unit of alcohol per week (*n* = 113), and the second group was comprised of those who consume 2 or more units of alcohol weekly (*n* = 33). In addition, cadets were divided into smokers (*n* = 13) and non-smokers (*n* = 133). According to the Shapiro-Wilk test, all continuous variables, except body mass index (BMI) and the Zung crude score, were not normally distributed. Concerning the interpretation of the Greek translation of the Zung depression scale crude score, a cut-off value of 45 was selected because it displayed the highest specificity and sensitivity (both exceeded 90 %) in the Greek population [14]. Cadets were categorized into low (<45) and high risk (≥45) groups for the presence of depression. The mean Zung crude score in our cohort was 43.32 (SD = 4.55); 42.8 (SD = 4.43) for female and 43.64 (SD = 4.6) for male cadets (*P* = 0.28, from *t*-test). We identified 57 cadets with a total Zung crude score of 45 or above, which represented 39 % of our study population: 21 females and 36 males. Univariate statistical analysis with Pearson’s chi-squared test did not reveal any statistically significant differences between the two groups regarding risk factors for the presence of depression such as gender, year of training, or academic performance. In addition according to analysis with Pearson’s chi-squared test, *t*-test and the Mann Whitney *U*-test, lifestyle characteristics such as smoking status, alcohol consumption, BMI or dietary habits did not differ between the two groups. Moreover, according to the Pearson correlation test, there was no statistically significant correlation between BMI and Zung crude score (*r* = −0.063, *P* = 0.45). A more in-depth statistical analysis was attempted with binary logistic regression analysis which included both established and potential risk factors but did not yield any statistically significant results. Tables 1 and 2 summarize the results of our statistical analysis.

**Table 1:** Risk factors for presence of depressive symptoms

Risk factor	Low risk^a^ [n(%^b^)]	High risk^a^[n(%^b^)]	P value from univariate analysis	P value from binary logistic regression	Total [n(%^b^)]
Gender			0.87^*^	0.244	
Male	55 (61.8 %)	36 (63.2 %)			91(62 %)
Female	34 (38.2 %)	21 (36.8 %)			55(38 %)
Academic performance					
High	17(19.1 %)	10 (18.5 %)	0.67^*^	0.396	27(18 %)
Average	57 (64 %)	34 (62.3 %)			81(64 %)
Low	15 (16.9 %)	13 (19.2 %)			28(18 %)
Year of training			0.45^*^	0.198	
1st	21 (23.6 %)	15 (26.3 %)			36(24.7 %)
2nd	34 (38.2 %)	16 (28.1 %)			50(34.2 %)
3rd	34 (38.2 %)	26 (45.6 %)			60(41.1 %)

^*^ *P* value from Chi-square test

^a^high and low risk for the presence of depression according to Zung self-reporting depression scale

^b^percentage within group

**Table 2:** Lifestyle characteristics of depression groups

Variable	Low risk^a^[n(%^b^)]	High risk^a^[n(%^b^)]	P value from univariate analysis	P value from binary logistic regression	Total [n(%^b^)]
Smoking			0.96^*^	0.892	
Yes	8 (9.0 %)	5 (8.8 %)			13 (9.0 %)
No	81 (91.0 %)	52 (91.2 %)			133 (91.0 %)
Vitamin supplementation			0.83^*^	0.934	
Yes	15 (16.9 %)	11 (19.3 %)			26 (18.0 %)
No	74 (83.1 %)	46 (80.7 %)			120 (82.0 %)
Energy drink use			0.93^*^	0.635	
Yes	12 (13.5 %)	8 (14.0 %)			20 (14.0 %)
No	77 (86.5 %)	49 (86.0 %)			126 (86.0 %)
Alcohol consumption			0.45^*^	0.258	
0-1 units	67 (75.3 %)	46 (80.7 %)			113 (77.0 %)
> 1 units	22 (24.7 %)	11 (19.3 %)			33 (23.0 %)
Daily coffee consumption (units) [median (IQR)]	1 (0.0-2.5)	2 (1.0-2.5)	0.32^**^	0.078	2 (1.0-2.3)
Daily fruit/vegetable consumption (servings) [median (IQR)]	2 (1–2)	2 (1–2)	0.28^**^	0.522	2 (1–2)
Weekly fish consumption (servings) [median (IQR)]	1 (1–1)	1 (1–1)	0.27^**^	0.173	1 (1–1)
BMI (kg/m^2^) [mean (SD)]	23.32 (2.19)	23.10 (1.87)	0.53^***^	0.285	23.23 (2.06)

^*^ *P* value from Chi-square test

^**^ *P* value from Mann Whitney *U* test

^***^ *P* value from *t*-test

^a^high and low risk for the presence of depression according to Zung self-reporting depression scale

^b^percentage within group

## Discussion

The aim of this study was to examine the prevalence of depressive symptoms in military medicine cadets, explore possible associations with several possible risk factors for depression and investigate the lifestyle characteristics of cadets with depressive symptoms. Military medicine undergraduates are a subpopulation of medical students and military personnel with unique characteristics: they are subject to a physically and psychologically intense lifestyle with demanding medical studies and arduous military training. According to our results, the overall depression symptom prevalence in our cohort was 39 %, which is comparable to the single study in the current literature examining a similar population (34.7 %) [8].

Depression in non-military medical students has been explored extensively. *Haldorsen*, et al. [5] document a similar depression rate (30.4 %), whereas *Honney* et al. [6] demonstrated an even higher rate (48.8 %). Other studies report a substantially lower point-prevalence (2.9 %-14.3 %) [3, 7].

Without doubt, the military environment has unique characteristics: austere discipline, rigid hierarchical structure, laborious physical training, strict rules, separation from family, and isolation from the familiar civilian setting for long periods of time [2]; such stressors may have a crucial role in the emergence of depressive symptoms [15]. The pathophysiological mechanism behind this link is complex; one theory speculates that following a stressor, there is a dysregulation of the hypothalamic-pituitary-adrenal axis, resulting in the hypersecretion of adrenal steroids, which contributes to neuron loss in the hippocampus, a brain region involved in the pathogenesis of depression [16, 17].

Multiple studies have investigated the prevalence of depressive symptoms in non-medical military trainees. For example, a cross-sectional study employing the self-rating depression scale (SDS) revealed a higher mean depression score in a cohort of 466 Chinese soldiers who had just completed their initial recruit training, compared to the general population [18]. In another study by *Iversen* et al. [19] the Patient Health Questionnaire 9 was administered via telephone interview in a sample of UK military personnel drawn from the phase 1 KCMHR military health study, half of whom were deployed during the active phase of the 2003 Iraq War; in that cohort the prevalence of any depressive syndrome and major depressive syndrome was 11 and 3.7 %, respectively. Moreover, in another cross-sectional study using the SDS, *Xiong* et al. [2] reported a depression rate of 25.2 % during field military training in a random sample of 1,220 Chinese soldiers. In that study, higher education level, shorter military service, origin from a city or town and health problems during military training were associated with depressive symptoms.

Alternatively, weak or exceptional academic performance as well as female gender and lower class year have been identified as risk factors for the presence of depression in non-military medical students [3, 7, 20]. However, we did not discover any differences in depression prevalence with academic performance, year of training or gender. *Güleç* et al. [8] previously reported an association between smoking and depressive symptoms in a sample of 690 Turkish military medicine cadets; more specifically, smokers were 2.2 times more likely to exhibit depressive symptoms than non-smokers. In our study, we did not observe an increased prevalence of smoking among cadets with depressive symptomatology. A possible explanation is the lack of necessary statistical power, given that only 13 cadets (9 % of our study population) were smokers.

*Chang* et al. [21] reported that only 24 % of non-military medical students with depressive symptoms used their school’s mental health counseling services. According to a recent report from the U.S., more than half of affected military personnel did not seek professional help despite the presence of mental health problems [22]. A systematic review of literature reported a high prevalence of anticipated stigma in military personnel with mental health problems but a poor association with help-seeking intentions and mental health service utilization. According to the authors, methodological issues in self-stigma measurement as well as intention-behavior gaps may account for that lack of association [4]. Nevertheless, the majority of evidence in civilians points to a negative association between stigma and help-seeking behavior [23]. As such, further studies within the military are needed to clarify this inconsistency. We must also note that evidence regarding the prevalence of depressive symptoms among commissioned officers, who hold key leadership positions, is scarce.

Our study has a number of limitations: first, our study population consisted only of cadets during their initial three years of medical training, who receive minimal clinical medical exposure compared to senior cadets of the latter three years. In addition, we did not employ a second depression screening tool to confirm our findings. Confirmation of the diagnostic status of any individual included in our study could not be performed due to the use of self-reporting of symptom severity. The majority of studies in the literature assessing depression prevalence among medical students utilize questionnaires, such as the Beck Depression Inventory or Patient Health Questionnaire 9. These screening tools, including the Zung, which was used in our study, are self-reporting depression scale questionnaires; the honesty of participants cannot be guaranteed. Therefore, another limitation of our study is the self-reporting nature of the administered questionnaire.

These screening tests may also overestimate the prevalence of depression; for instance, a large study utilizing the Mini International Neuropsychiatric Interview reported a much lower rate (2.9 %) of depression in medical students [7]. However, time restrictions and cost effectiveness make self-reporting questionnaires acceptable tools for providing a crude picture of depression prevalence, and may guide further interventions.

## Conclusions

We report a relatively high prevalence of depressive symptoms in a sample of military medicine cadets. We could not identify any related risk factors in our cohort. Although this percentage may not represent the true prevalence of depression, it demonstrates the need for further investigation. Despite the limitations of our study, our findings indicate that military medicine cadets who are exposed to a demanding medical and military environment are a group at risk for the emergence of depression. This finding emphasizes the importance of depression screening in every military academy and the development of mechanisms to ensure an efficient intervention in a timely manner.

## Abbreviations

BMI: Body mass index
GPA: Grade point average
MMPI: Minnesota Multiphasic Personality Inventory
SSAS: Hellenic Military School of Combat Support Officers (Greek Spelling: Stratiotiki Scholi Axiomatikon Somaton)
